# Negotiating pricing and payment terms for insurance covered mHealth apps: a qualitative content analysis and taxonomy development based on a German experience

**DOI:** 10.1186/s13561-024-00558-8

**Published:** 2024-10-04

**Authors:** Bettina Freitag, Leonard Fehring, Marie Uncovska, Alexandra Olsacher, Sven Meister

**Affiliations:** 1 Health Care Informatics, Faculty of Health, School of Medicine, Witten/Herdecke University, Alfred-Herrhausen-Straße 50, Witten, 58455 Germany; 2 Faculty of Health, School of Medicine, Witten/Herdecke University, Alfred-Herrhausen-Straße 50, Witten, 58455 Germany; 3 Department Healthcare, Fraunhofer Institute for Software and Systems Engineering, Emil-Figge-Straße 91, Dortmund, 44227 Germany; 4 Helios University Hospital Wuppertal, Gastroenterology, Heusnerstraße 40, Wuppertal, 42283 Germany

**Keywords:** MHealth, DiGA, Pricing, Reimbursement, Taxonomy development, Qualitative content analysis

## Abstract

**Background:**

Germany was the first country worldwide to offer mobile digital health applications (mHealth apps, “DiGA”) on prescription with full cost coverage by statutory health insurances. Especially statutory health insurances criticize the current pricing and payment regulations in Germany due to “free and non-transparent” pricing in the first year and lack of cost use evidence. The study consists of two parts: The first part evaluates interests of digital health application providers and statutory health insurances in Germany to identify overlaps and divergences of interests. The second part includes the development of a comprehensive pricing and payment taxonomy for reimbursable mHealth apps in general.

**Methods:**

Both parts of the study used the input from 16 expert interviews with representatives of digital health application providers and statutory health insurances in Germany. In part one the authors conducted a qualitative content analysis and in part two they followed the taxonomy development process according to Nickerson et al. (2013).

**Results:**

A value based care model is expected to bring the greatest benefit for patients while statutory health insurances welcome the idea of usage based pricing. The final pricing and payment taxonomy consists of four design and negotiation steps (price finding, payment prerequisites, payment modalities, composition of negotiation board).

**Conclusions:**

As healthcare resources are scarce and thus need to be optimally allocated, it is important to implement pricing and payment terms for reimbursable mHealth apps that result in the greatest benefit for patients. To the best of the authors’ knowledge, there has been no structured study yet that examines alternative pricing strategies for reimbursable mHealth apps.The developed pricing and payment taxonomy for reimbursable mHealth apps serves as planning and decision basis for developers, health policy makers and payers internationally.

**Supplementary Information:**

The online version contains supplementary material available at 10.1186/s13561-024-00558-8.

## Background

**Fig. 1:**
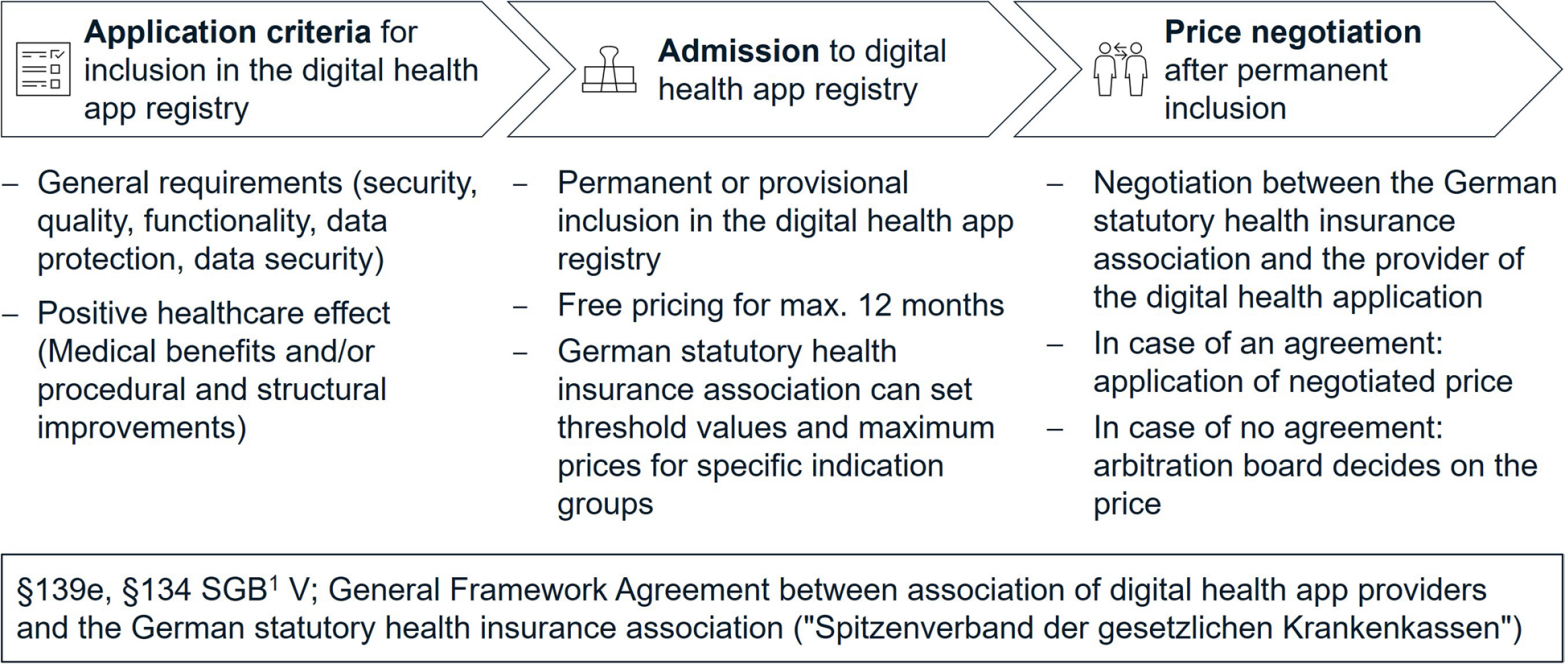
Overview of the current price finding process for DiGAs in Germany. 1: "Sozialgesetzbuch" (German law with regulations for statutory health insurances)

Pricing decisions for mobile health (mHealth) applications (apps) are challenging due to varying cost structures of mHealth apps and a rapid change in scale compared to traditional healthcare technologies [1]. A study surveying 1.051 participants in Germany found that although the general willingness to use mHealth apps is high, only 27% of all respondents were willing to pay for mHealth apps [2]. In the United States another study concluded that 77% of all participants use free mHealth apps and would pay one dollar or less for additional unique functions and features [3]. Thus, Germany was the first country worldwide to allow prescription of certified mHealth apps by physicians with cost coverage by statutory health insurances (SHIs). These apps are called “Digitale Gesundheitsanwendungen” (DiGA) or in English “digital health applications”. Existing research shows that DiGAs have significant potential to facilitate patient access to health care and are a valuable complement to existing therapies [4–8]. The recently introduced Digital Health Care Act (“Digitale-Versorgung-Gesetz”) in Germany aims to promote the implementation of digital health applications [9]. To gain prescribing and reimbursement status, DiGAs must undergo a comprehensive certification process (see Fig. 1) and provide scientific evidence of effectiveness through clinical trials and compliance with general requirements (e.g., data protection, safety, interoperability).

After being listed in the DiGA registry, DiGA providers are free to set a price for the first year given a maximum price limit per indication group [10]. Maximum price limits as of September 2022 range from ~ 219 euros (90 day prescription) for diseases of the musculoskeletal system to ~ 742 euro (90 day prescription) for diseases of the nervous system [11]. After one year, the German statutory health insurance association “GKV Spitzenverband” (GKV-SV) and DiGA providers negotiate the price for the app [12]. In the event of a disagreement, an arbitration board steps in. This arbitration board consists of an impartial chairperson, two other impartial members and two representatives of the GKV-SV and the association of DiGA providers (§134 Abs. 3 SGB). Prior experiences showed that the board must become increasingly active to achieve consensus in the price negotiations [13]. The GKV-SV criticize the free pricing regulation in the first year, especially in case of provisionally accepted DiGAs that lack final proof of the positive health care effect through clinical trials [14].

To optimize current pricing regulations towards more efficient and patient-centered care, current research discusses mainly two alternative pricing strategies for DiGAs. On the one hand, value based care pricing models consider the effectiveness of DiGAs where SHIs would pay a certain amount depending on the positive reported health effect of the app [15–18]. Patient questionnaires are one possibility to operationalize value based care pricing models because the effectiveness could be measured based on patients’ experiences with the app [19]. On the other hand, patient adherence is another concept, where SHI would only reimburse DiGAs that are actually used by patients, i.e. this pricing model should avoid that SHIs pay for apps that are for example only downloaded but never used [20]. Alternative and internationally common pricing strategies exist in the area of pharmaceuticals, e.g., external reference based pricing models, where prices are derived based on similar products on the market [21–23] or managed entry agreement based pricing models which are based on individual negotiations between payers and pharmaceutical companies [24].

To the best of our knowledge, there has been no scientific research yet that assessed the different pricing interests of both DiGA providers and SHIs and that has structurally examined different potential alternative pricing and payment terms for reimbursable mHealth apps. In order to close this research gap and ensure a patient-centric care and fair allocation of resources, we aim to answer the following research questions:

Q1: What are the different pricing interests and overlaps of interest of DiGA providers and SHIs in Germany?

Q2: What is a suitable taxonomy to describe pricing and payment terms for reimbursable mHealth apps?

## Methods

The goals of this paper were to develop an internationally applicable taxonomy of pricing and payment terms for reimbursable mHealth apps, to derive advantages and disadvantages of selected pricing models and to identify overlaps of interest in terms of pricing between DiGA providers and SHIs. To fulfill these goals, we pursued a twofold research approach: On the one hand, we followed the taxonomy development process according to Nickerson et al. [25] in order to derive a pricing and payment taxonomy for reimbursable mHealth apps. We conducted a scoping review, held interdisciplinary scientist panels and performed expert interviews with representatives of both SHIs and DiGA providers in Germany which all served as underlying data for the taxonomy development process. On the other hand, we used insights from the expert interviews with SHIs and DiGA providers to identify perceived challenges and opportunities of the existing pricing model, to discuss opposing opinions on alternative pricing strategies and to derive common interests between SHIs and DiGA providers.

### Expert interviews

All interviews were conducted based on a pre-developed semi-structured interview guideline consisting of open and semi-quantitative questions structured in four interview sections: a) Introduction and discussion of challenges and opportunities of DiGA pricing regulations b) Discussion of alternative pricing models c) Evaluation of alternative pricing models d) Further ideas and forecast of general DiGA development. After approval by the Ethics Committee, the interview guideline was prototyped and minorly adjusted in terms of structure (see Additional file 1 for complete final interview guideline). We conducted 16 expert interviews in two focus groups, one consisting of representatives of DiGA providers and the second consisting of representatives of SHIs. The interviews per focus group were split equally and hold between May and September 2022, lasting from 30 to 90 min. We identified the 30 largest SHIs in Germany by number of insured persons and all DiGA providers that were permanently or provisionally listed in the DiGA registry as of 15.03.2022 (official start date of the research after positive ethics approval has been received). Potential organizations were contacted by email, phone and/or the platform LinkedIn. Since the response rate of DiGA providers was too low to reach the desired sample size, we also included mHealth app providers that are in the process of being listed in the DiGA registry as well as a federal SHI association that were recommended in a snow-ball like principle by other experts in the DiGA providers’ expert group. We interviewed 1 permanently included DiGA provider, 2 provisionally listed DiGA providers and 5 potential DiGA providers that are currently in the process of being listed.

All interviews were conducted virtually and transcribed according to the transcription system of Dresing and Pehl [26] using the transcription software Trint. We systematically analyzed the interview material according to the content-structuring qualitative content analysis of Kuckartz [27] using the software MAXQDA2022. We ran two coding cycles to synthesize the large amount of data to key patterns, the first coding category was derived from our interview guideline (deductive approach), which we further refined in the second coding cycle by incorporating the findings from the interviews (inductive approach). The consolidated criteria for reporting qualitative studies (COREQ) according to Tong et al. [28] can be found in Additional file 2.

The interviews also contained a semi-quantitative part to examine selected pricing models given pre-defined evaluation criteria. This analysis was conducted in Microsoft Excel and Power BI based on the evaluations in the interviews. One representative of the DiGA providers focus group did not participate in this survey, i.e. we had overall 7 respondents of the DiGA providers’ and 8 respondents of the SHIs’ focus group. To test for significance of our results, we conducted a Mann–Whitney-U-Test, which is used especially for small samples to test whether the central tendencies of two independent samples are different. For each pricing model and evaluation criteria, we tested whether the average opinion of the DiGA providers’ expert group is significantly different to the average opinion of the SHIs’ expert group at a significance level of 5%.

### Taxonomy development

We followed the taxonomy development model according to Nickerson et al. [25]. The taxonomy development process combines a deductive (“conceptualization”) with an inductive (“empiricism”) approach such that both strategies can be used in an iterative manner to develop a “useful taxonomy with mutually exclusive and collectively exhaustive characteristics” [25]. Literature describes this approach also as abductive, i.e. developing theoretical insights from data using an added element of human thinking [29]. The approach is consistent with the “generate/test cycle” in the design science research described by Hevner et al. [30], where so called artefact design is explained as a continuous search process.

**Fig. 2:**
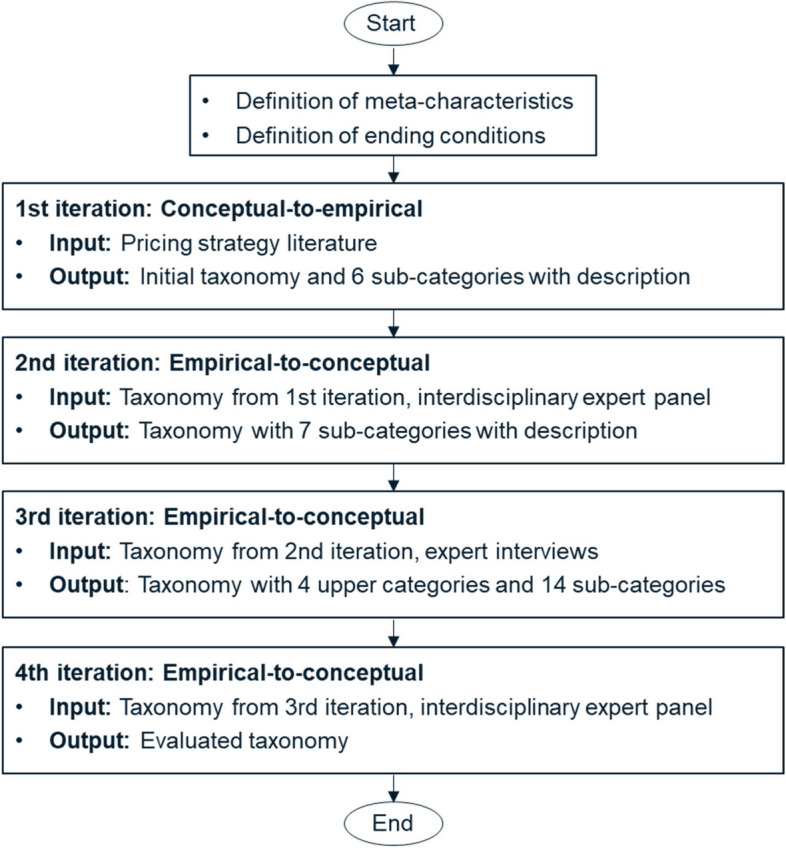
Taxonomy development process

First, we defined meta characteristics that “serve as the basis for the choice of characteristics in the taxonomy” [25]. Since we expect our taxonomy to be used by decision makers and practitioners with respect to the design of mHealth app pricing guidelines, we defined the underlying meta characteristic as *dimensions that are relevant for the design and negotiation of mHealth pricing and payment terms*. Each dimension and characteristic of our pricing and payment taxonomy for reimbursable mHealth apps must rely to this principle, which helped us to identify and structure relevant dimensions. Second, we selected six subjective and four objective ending conditions of the ones suggested by Nickerson et al. [25]. Overall, we ran four iterations to develop our pricing and payment taxonomy for reimbursable mHealth apps. Figure 2 summarizes our methodological approach.

#### 1st iteration (conceptual-to-empirical)

**Fig. 3:**
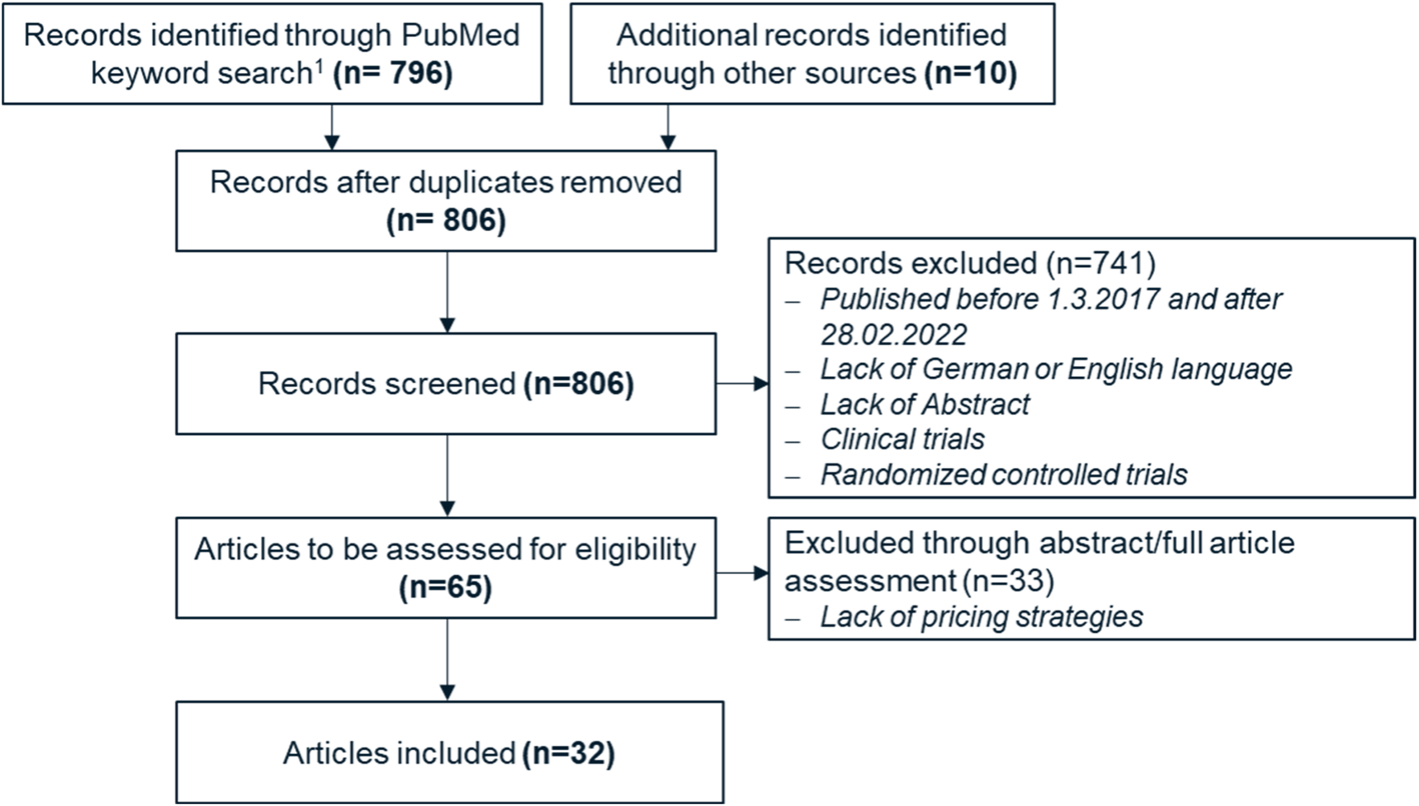
Scoping review in accordance with PRISMA-ScR. 1: PubMed keyword search based on keywords in abstract or title: (“pricing strategies” OR “pricing strategy” OR “pricing” OR “reimbursement” OR “payment”) AND (“mHealth apps” OR “DiGA” OR “digital health applications” OR “pharmaceuticals”)

Our first iteration was conceptual-to-empirical where we conducted a structured scoping review [31] to identify already existing concepts with regards to pricing, payment and reimbursement applied for mHealth apps or pharmaceuticals in literature. Figure 3 shows the flow diagram for the selection of sources of evidence using an extension for scoping reviews of the preferred reporting items for systematic reviews and meta-analyses (PRISMA-ScR) [32]. To be included in the scoping review, articles must be listed in the ‘PubMed’ database and published between 01.03.2017 and 28.02.2022. The article must be in English or German language and must include an abstract. The article types “clinical trials” and “randomized controlled trials” were excluded from the analysis since their main focus lies on the proof of effectiveness and security of new treatment options rather than suggestions for pricing and reimbursement schemes.

Abstract and full-text assessment was then conducted to filter those articles that incorporated pricing strategies. 32 research articles met our inclusion criteria and were used as basis to derive seven pricing strategies for mHealth apps based on existing concepts. Those pricing strategies served as input for our next iteration cycle.

#### 2nd iteration (empirical-to-conceptual)

The next iteration was empirical-to-conceptual by holding an interdisciplinary scientist panel consisting of physicians, economists, computer scientists and health insurance experts who systematically evaluated the results of the scoping review and checked whether there are other pricing models that were not mentioned in the literature search. The scientist panel added one pricing model which they named user experience based pricing model, where the price of a mHealth app would depend on a user rating with respect to user experience. This scientist panel also designed the semi-structured interview guideline as basis for the already mentioned expert interviews.

#### 3rd iteration (empirical-to-conceptual)

We used our expert interviews of DiGA providers and SHIs to further refine our mHealth app pricing and payment taxonomy. We added four overarching design and negotiation steps and seven design choices based on the opinions and ideas of our experts.

#### 4th iteration (empirical-to-conceptual)

To empirically evaluate the mHealth app pricing and payment taxonomy, we held the interdisciplinary scientist panel from the 2nd iteration again to discuss the revised mHealth app pricing and payment taxonomy including all newly added elements. One author served as moderator. Throughout this scientist panel discussion, we evaluated all design and negotiation steps and design choices and only fine-adjusted descriptions. At the end of this iteration cycle, both our objective and subjective ending condition were met: The mHealth app pricing and payment taxonomy was “concise”, “robust”, “comprehensive”, “extendible” and “explanatory” (subjective ending conditions, [25]). Regarding the objective ending conditions, no category was merged with any other category or split into multiple categories, there is at least one sub-category per category, no new categories or sub-categories were added, every category and sub-category is unique and not repeated (objective ending conditions, derived from [25]).

After the 4th iteration of our taxonomy development process, we came up with our taxonomy for the design and negotiation of reimbursable mHealth app pricing and payment decisions.

## Results

### Expert interviews

#### Perceived opportunities and challenges of the DiGA implementation in Germany

By coding our expert interview material, we found that experts of both groups (3/8 DiGA providers, 4/8 SHIs) highlight the existence of the legal framework of fully covered mHealth solutions in Germany positively and think that it fosters digitalization of the German health system. 3/8 SHIs mention the establishment of a digital and automatic reimbursement process positively, i.e. invoices no longer have to be submitted in paper form. Whereas DiGA providers emphasize the existence of the fast-track-procedure and in particular the possibility of free pricing in the first year (3/8 DiGA providers), one SHI rates the recent implementation of maximum price limits per indication positively. Besides the pricing topic, we found that DiGA providers value the exchange with the German Federal Institute for Drugs and Medical Devices (“Bundesamt für Arzneimittel und Medizinprodukte” (BfArM)) due to helpful suggestions for improvement and a profound review of the DiGA in development.

When looking at the key challenges, DiGA providers see regulatory requirements as a major hurdle resulting from increasing and changing requirements or the interpretation of those. Experts from both groups had negative experiences with arbitration hearings if there was no price agreement in the negotiations. For example some reported that they either had the feeling that the outcome was dependent on their counterparts, that there was no will to reach an agreement or that discussions seemed to be political.

#### SHIs perceive DiGAs as too expensive and scientific evidence not reliable

The coding of the expert interviews revealed that especially SHIs see the general pricing conventions critical, for example when prices have doubled or tripled, although those apps have been previously offered under individual contracts between DiGA providers and SHIs (so-called “selective contracts” in the German health system) or if DiGAs are not actually used by patients. SHI expert 8 emphasized “I think it is always important to understand that it is not the money of the health insurance company, it’s the money of the general public in Germany”. SHI expert 3 perceive DiGA prices as “arbitrary” and SHI expert 7 calls them “outrageous” and “subsidies for DiGA providers”. SHIs also criticize the current regulatory framework regarding necessary evidence for being listed in the DiGA registry. Especially the fact that DiGAs that are provisionally listed in the DiGA registry still lack the final scientific proof of evidence but are refundable to a self-selected price (considering the maximum price regulation) was one major pain point. In the case of currently permanently included DiGAs, SHI experts questioned the robustness of studies, e.g., the sample sizes, the study design and the outcome measurement (in line with [33]).

#### DiGA providers criticize the cost orientation of the German healthcare system

On the other side, DiGA providers criticize the strong cost orientation of the German healthcare system (“This is pure ownership thinking, pure political thinking, economic-political thinking and does not have anything to do with improving care for patients or innovating the German healthcare system—this is not in the interest of SHIs.” – DiGA provider expert 1). Hence, some DiGA providers hypothesize that SHIs do not promote DiGAs due to high perceived cost for the healthcare system.

#### SHIs’ and DiGA providers’ perspective on alternative pricing strategies

**Fig. 4:**
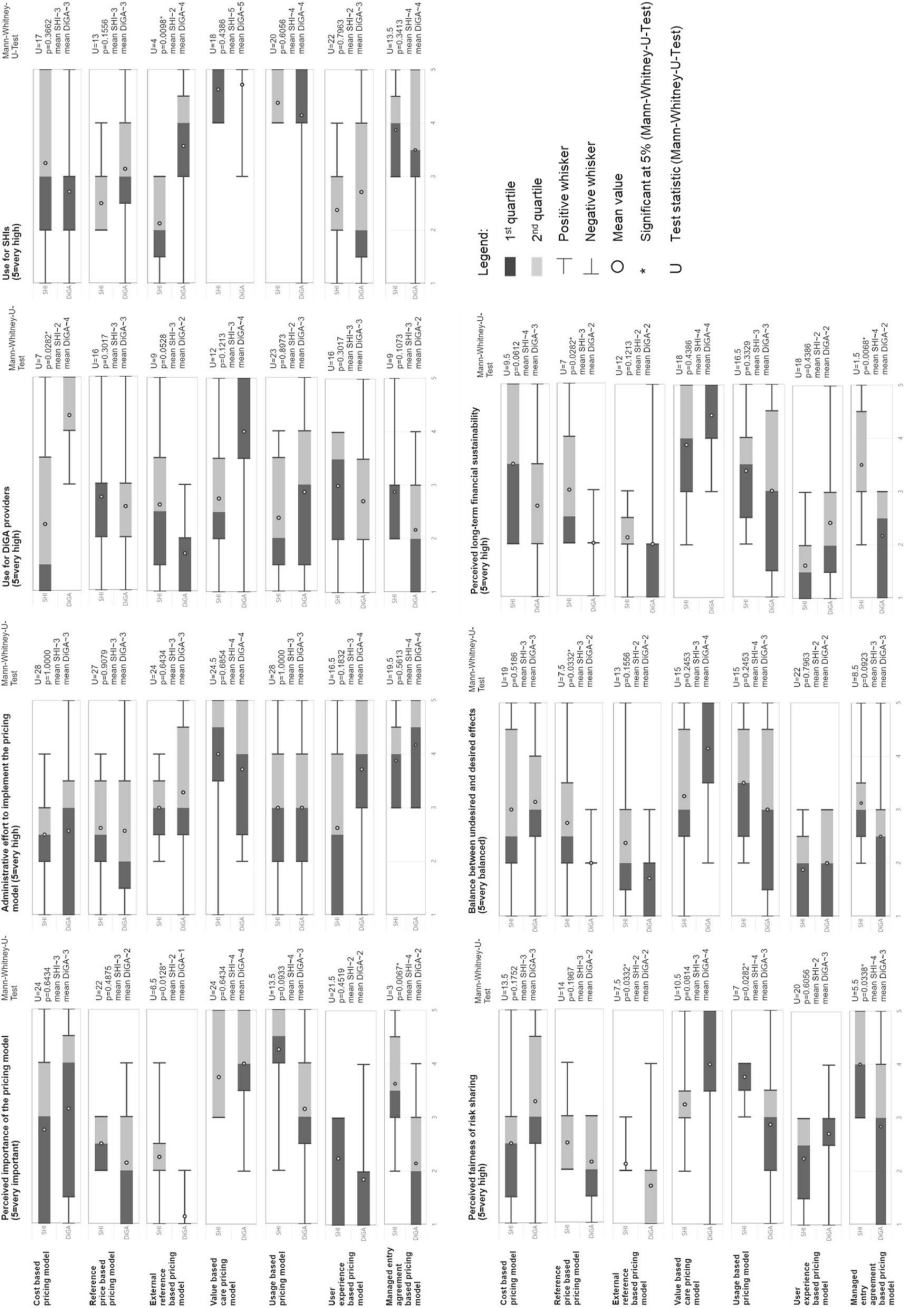
Pricing model evaluations by SHI and DiGA provider experts in 2nd iteration of taxonomy development

To overcome the described challenges with respect to DiGA pricing, we structurally examined seven alternative pricing strategies in our expert interviews. Those were a cost based, a reference based, an external reference based, a value based care, a usage based, a user experience based and a managed entry agreement based pricing model. A detailed definition, overview of mentioned advantages and disadvantages of suggested pricing models is illustrated in Table 1. The results of the semi-quantitative evaluation can be found in Fig. 4.

**Table 1:** Overview of pricing models after 2nd iteration. This table shows advantages and disadvantages of the pricing strategies discussed by SHI and DiGA provider experts in the 2nd iteration of the taxonomy development process

Pricing model [Design choice^1^]	Description	Advantages	Disadvantages
Cost based pricing model [cost based pricing model (with/without profit markup)]	In a cost-based pricing model, the price of a DiGA is based on the individual development and operating costs of the app.	• Creates transparency about costs and promotes understanding on the side of SHIs • Can help to promote innovation on a short-term basis	• Difficulty to operationalize and guarantee identical measurement procedures (e.g. comparable underlying assumptions concerning usage duration, discussion about profit margin, know-how to evaluate cost structure on the side of SHIs) • Price model can be susceptible to manipulation, if cost structure is reported incorrectly • App effectiveness is not considered
Reference price based pricing model [indication group based pricing model]	In a reference price-based pricing model, average prices are set per indication group and applied equally to all DiGAs in the respective indication group.	• Creates plannable income for DiGA providers/plannable expenses for SHIs • Creates a basic framework and price transparency for indication groups	• App developers are not incentivized to develop superior products as same price is paid independent of functionalities and user friendliness • Difficulty to form indication groups and agree on average prices • Price model can be susceptible to manipulation if DiGA providers make (illegal) price arrangements • App effectiveness is not considered
External reference based pricing model [external reference based pricing model]	In an external reference based pricing model, an international benchmark price/comparison price is derived based on an application already existing in the market.	• International prices can lead to cost-efficient prices for the healthcare system	• Difficulty to compare different market specifics and regulatory requirements (e.g., different development cost, marketing cost, specialties of the market, organization and specifics of local healthcare systems, patient populations etc.) • High effort to operationalize pricing model in a fast changing and dynamic environment (e.g., find similar measurement parameters, deal with quality differences and manipulation attempts etc.) • App effectiveness is not considered
Value based care pricing model [value based care: real-world evidence]	In a value based care pricing model, the price of the app is based on the actual medical benefit for the patient. Payment is only made if the treatment is actually successful.	• Considers patient outcomes of the DiGA • Offers ‘right’ incentives for DiGA providers • Offers basis for other medical care areas	• Difficulty to operationalize (e.g. measurement of DiGA effectiveness, interdependencies to other medical treatments, difficulty to implement pricing model in IT systems) • Limitations concerning data protection and data security
Usage based pricing model [usage based]	In a usage based pricing model, the price of the app is based on the usage duration per patient.	• Usage data is generally available • Guarantees that app is only paid if it is used • Eventually there might be a positive effect between app usage and app effectiveness and user experience of the app	• Difficulty to operationalize, e.g., necessary usage time mighty vary per indication group and app, specialties with apps with hardware • Difficulty to define price staggering, e.g., usage as a hygiene factor, measurement at follow-up prescriptions • Usage is no guarantee for app effectiveness • Can cause misincentives for DiGA providers (e.g., development of apps that are often used but have no effectiveness) • Limitations due to data protection, there could occur the feeling that SHIs monitor their insurees
User experience based pricing model [user experience based]	In a user experience based pricing model, the price of the app is based on the average patient ratings in terms of user experience of the app.	• Increase in therapy compliance if user experience of the app is good	• No direct connection to the medical evidence • Can cause misincentives for DiGA providers, e.g., development focus on fun factor except of medical effectiveness • Pricing model is susceptible to manipulation, e.g., employees of DiGA providers could write app assessments themselves/rate apps • Pricing model is dependence of patients’ input • Could be perceived as redundant parameter in comparison to other pricing models, i.e., if user experience is unsatisfactory, app would not be used/would have no effect • Variance between age groups in terms of user experience needs to be addressed, e.g., digital natives versus digital immigrants
Managed entry agreement based pricing model [individual health insurance company]	In a managed entry agreement based pricing model, DiGA providers and SHIs negotiate a framework agreement that is subject to certain conditions.	• Enables individual negotiations with individual conditions • Broadens applications areas, e.g., integration with electronic health record, telemedicine, medical supervision of the treatment etc.	• SHIs have high negotiation power, might be difficult for DiGA providers to ‘survive’ in a price competition and deal with regional dependencies to SHIs • High bureaucratic effort to individually negotiate, could result in difficulties for DiGA providers and smaller SHIs • Can be detrimental for innovation if DiGA providers rest on past negotiations and do not further invest in innovation • Limitation of the DiGA reach (i.e. not all insurees have access to all DiGAs)

^1^Design choice of the final taxonomy for the design and negotiation of reimbursable mHealth pricing and payment terms that is most similar to the presented pricing models in the expert interviews is mapped

When asking each expert in the interviews for their top 3 pricing model, we found that the value based care pricing model and usage based pricing models were mentioned most by all experts.

- *Value based care pricing model*: The value based pricing model is considered as the fairest risk sharing model by both SHIs and DIGA providers because it incentivizes positive health outcomes or structural health system improvements. All experts agreed that a value based care pricing model would cause the “right” incentives for DiGA providers since this model motivates DiGA providers to maximize the medical outcome of the app. This is also reflected in the evaluation criteria “use for DiGA provider” and “use for SHIs” since both expert groups see a high use for themselves. Interestingly, SHIs think on average that the use of the pricing model for DiGA providers is low to medium (note that the difference in opinions is not significant according to the conducted Mann–Whitney-U-Test). In addition to this, the value based care pricing model is seen as the most sustainable one. Nevertheless, this pricing model comes with one downside: Experts of both groups saw difficulties and high bureaucratic efforts in operationalizing this pricing model, e.g., how to quantify and measure the effectiveness of a DiGA in a given period and who is responsible for the measurement. (“The optimum would be to have a double evaluation – one from the patient and one from the physician.”—DiGA provider expert 7).
- *Usage based pricing model*: SHIs consider the usage based pricing model to be the most important since this pricing model would guarantee that SHIs only pay for DiGAs which are used on a regular basis by patients. SHI experts reported that past analysis have shown that a considerable number of activation codes were not redeemed or that users have signed up once and then only used the app a few times or never. Consequently, both expert groups agree that the use of this pricing model for SHIs is on average between high and very high. Experts of both groups also critically discussed the concrete operationalization of this model, considering whether a minimum required use time (e.g., 30 min per day spent on the application) or usage staggering levels (e.g., payment after download, after log-in, after a pre-defined number of usages etc.) should be implemented. DiGA provider expert 8 stated that “if SHIs find finally their peace of mind by implementing usage based payment, we can live with it.”
- *Cost based pricing model*: DiGA providers estimate the greatest use for themselves when having the cost based pricing model due to low implementation efforts and full security concerning development cost coverage. Interestingly, SHIs see rather low use for DiGA providers (difference is significant, *p* = 0.0282). SHI expert 4 states that “The risk of this pricing model is that DiGA providers could drive up development cost to later justify high app prices.”
- *Reference based pricing model*: Both expert groups categorize the reference based pricing model as less important and more as transitional pricing model in the initial phase due to the difficulty to compare different DiGAs within one indication group.
- *External reference based pricing model*: The external reference based pricing model is classified as less important by our experts and less balanced in terms of desired and undesired effects. Both expert groups evaluate the external reference based pricing model lowest in terms of fair risk sharing due to external dependencies that are beyond the own sphere of influence. DiGA providers think this pricing model is of rather high use for SHIs while SHIs themselves see rather low use in it (difference is significant, *p* = 0.0098).
- *User experience based pricing model*: It is striking that especially the user experience based pricing model is estimated as rather unbalanced in terms of desired and undesired effects by both experts groups. Experts stated that they see a risk of manipulating this pricing model for example by giving fake reviews. DiGA providers could for example be incentivized to design a user-friendly app that is fun to use but has no medical effectiveness (“I understand the concern of payers and their aversion that they say, we do not want to pay for app icons on smartphones”—DiGA provider expert 1). Overall, user experience based pricing model are classified as less important by our experts and SHIs evaluate the user experience based pricing model as the least financial sustainable model.
- *Managed entry agreement based pricing model*: SHIs find the managed entry agreement based pricing model (*p* = 0.0428) significantly more important than DiGA providers. DiGA providers emphasize the great implementation effort and describe this pricing model as a step back compared to the current pricing model.

Among all evaluation criteria, there is the highest number of significant differences for the *perceived fairness of risk sharing*, i.e. for the external reference based pricing model, usage based pricing model and managed entry agreement based pricing model perceived fairness of risk sharing is significantly valued differently by both expert groups.

#### Requirements for future DiGA pricing models

Another finding of the expert interviews were general requirements that should be incorporated in the design of future pricing models. First, we found that the DiGA pricing model should be embedded in the overall patient care including physician therapy support. Physicians prescribe a DiGA for a special indication and should also monitor and support patients while using them, where a DiGA pricing model could also create incentives for (apart of the current billing figures for prescribing a DiGA) according to the expert opinions. Second, the DiGA pricing model should be dynamic and able to quickly adapt to changing market, technological or regulatory requirements. One DiGA provider also stated that the DiGA pricing model should enable innovation and hence offer “necessary freedom (DiGA provider expert 8)” for further technical development of the app. Third, we found that it is essential to create systemic conditions to enable the implementation of innovative pricing models, e.g. operationalization mechanisms for value based care pricing models.

### Results of taxonomy development: The developed pricing and payment taxonomy for reimbursable mHealth apps

After the 4th iteration of our taxonomy development process, we came up with a revised taxonomy for the design and negotiation of reimbursable mHealth app pricing and payment decisions. We introduced overarching design and negotiation steps, added dimensions and fine-adjusted descriptions. As we found in our expert interviews, that most of the experts favor a combination of the different pricing models, we renamed the term “pricing model” in “design choices”, indicating that a combination of several factors is possible.

We introduced an additional level of detail to categorize different pricing models (“Steps/elements of mHealth pricing and payment terms”). We differentiated our final pricing and payment taxonomy for reimbursable mHealth apps in 4 steps/elements: The first category “price finding” lists design choices that can determine the actual price height of a reimbursable mHealth app. The second category “payment prerequisites” lists various conditions that could determine under which circumstances payers actually pay for a reimbursable mHealth app (e.g., usage time, app effectiveness, etc.). “Payment modalities” describe the different accounting options for a reimbursable mHealth app indicating whether payers reimburse mHealth app developers per single patient or per fixed negotiated amount independent of the actual number of patients using the app. The fourth category “Negotiation board” specifies the actual stakeholder negotiating the above mentioned dimensions.

The suggested pricing and payment taxonomy for reimbursable mHealth apps can be found in  Table 2.

**Table 2:** Taxonomy for the design and negotiation of pricing and payment terms of reimbursable mHealth apps

Steps/elements of mHealth pricing and payment terms	Design choices	Description
Price finding	Cost based pricing model (with/without profit markup)	The price of a mHealth app is based on the individual development and operating costs of the app, plus a possible profit markup for the developer.
	Indication group based pricing model	Average prices are set per indication group and applied equally to all mHealth apps in the respective indication group, regardless of the actual functional scope or scientific evidence of the app.
	External reference based pricing model	A national, EU-wide or international benchmark (comparison) price is derived based on a comparative mHealth app already existing in the market that is similar in terms of functional scope and therapy effectivity.
	Analog-equivalent based pricing model	The cost of a comparable therapy is used for setting the price for the mHealth app, e.g., the costs of physical therapy sessions are used as proxies for pricing the mHealth app that could replace the physical therapy.
Payment prerequisites	Value based care: Real-world-evidence	The payment depends on the actual realized and measured medical benefit for the patient or procedural and structural improvements. Different levels of reimbursement can be triggered by different target achievement levels per patient or age-group.
	Value based care: Scientific evidence	The payment depends on treatment success for the patient or positive procedural and structural improvements demonstrated in the clinical trial study. Different levels of reimbursement can be defined by different target achievement levels per patient or age-group.
	Usage based	The payment is based on the patient’s usage time and/or usage frequency of the mHealth app. Different levels of reimbursement can be triggered by pre-defined threshold values (e.g., x times per month) or usage conditions (e.g., differentiation whether app has been downloaded, patient has registered, patient has a follow-up prescription).
	User experience based	The payment is based on the average patient ratings in terms of the user experience of the mHealth app. Different levels of reimbursement can be triggered by pre-defined user experience scores (e.g., star ratings in app stores).
Payment modalities	Per prescribed unit	Payers pay the negotiated price for the prescribed mHealth app per patient individually once the payment prerequisites are fulfilled.
	Per prescribed unit with capping	Payers pay the negotiated price for the prescribed mHealth app per patient individually until a defined maximum price once the payment prerequisites are fulfilled, e.g. as soon as a defined threshold value of prescriptions is reached payments are capped.
	Per prescribed unit with volume discount	Payers pay the negotiated price for the prescribed mHealth app per patient individually under consideration of a volume discount once the payment prerequisites are fulfilled.
	Fixed price	Payers pay a total fixed price to the developer independent of the number of prescribed units once the payment prerequisites are fulfilled.
Negotiation board	Representation board of payers	A representation board of payers negotiate the mHealth app’s price, payment prerequisites and modalities with individual developers (similar to current German pricing and payment model).
	Individual health insurance company	Individual developers and individual payers negotiate a framework agreement that sets the price, payment prerequisites and modalities (similar to managed entry agreement pricing model and selective-contract arrangements).

## Discussion

### Lack of cost use effectiveness analysis for DiGAs

The perceived high DiGA prices from the perspective of SHIs identified in our expert interviews are widely discussed in Germany at the time of writing. On average, the DiGA prices are about 400 euros for 90 days, ranging from 119 euro for a one-time license and 952 for 90 days in the first year (as of 30.09.2022). From the perspective of SHIs those prices are “arbitrarily set” and bear little relation to the reimbursement of conventional medical care. In addition, prices must not be disproportionate to the positive medical effect achieved or with comparable applications in the free market [11, 34]. Representatives of SHIs therefore claim that economic efficiency of DiGAs should be examined as one part of the admission procedure [34]. In the general context of health care evaluation this idea is also supported by Garrison et. al [35] suggesting that reimbursement decisions should be built on incremental costs and benefits of health care technologies provided in a cost effectiveness analysis. In the context of pharmaceuticals, cost effectiveness analysis are discussed as part of a broader multi criteria decision analysis (MDCA) to improve decision making within pricing and reimbursement processes [36, 37]. MCDA analysis can be either used as a hard decision factor for reimbursement if a specific threshold value is met or as supportive method to guide the final reimbursement decision [37]. Overall, the goal should be to “get the right (effective and cost effective) care to the right patients in the right setting at the right time” at an affordable price [38].

### Mutual understanding and transparency as basis for the price building process

Among all evaluation criteria, we found the highest number of significant difference in perceived fairness of risk sharing. We argue that it is especially difficult to find pricing and payment terms that are perceived as “fair” for all involved parties. Experts from our SHIs’ group explained that it is essential that the DiGA pricing and payment model enables innovation (“We must ensure that the pricing model does not kill innovation”—SHI expert 5). DiGA providers are faced with newly introduced maximum price limits and significant price reductions of 50 percent on average [14] after the price negotiations. We hypothesize that this price risk – in addition to the sales volume risk –could discourage DiGA providers from pursuing the official DiGA admission process and encourage selective contracting. Selective contracts are individual contracts between one or more SHIs, service providers and insurees in which services outside the scope of standard care can be agreed upon. This hesitation attitude is in line with existing work, stating that potential DiGA providers wait for first results of price negotiations and the associated market penetration opportunities before introducing further products in the DiGA fast track procedure [39]. Expert interviews revealed SHIs’ assumptions that DiGA providers deliberately choose their prices high in the first year in order to have a better negotiating position afterwards. Nevertheless, Greß et. al [40] found no evidence that DiGA providers have continuously increased their market prices to improve their negotiation position.

### Comparison to established price mechanisms in the German healthcare system

The pricing and reimbursement model for DiGA in Germany represents an innovation within the German healthcare system. Traditionally, healthcare services in Germany have been reimbursed through the following established mechanisms: Predominant in the outpatient sector, SHI reimburse a defined amount of money for each individual service being performed, e.g. for consultations or tests. Prices for those services are negotiated by a national evaluation committee and listed in the uniform evaluation standard for medical services in Germany (“Einheitliche Bewertungsmaßstab (EBM)”) [41]. Diagnosis-related groups are primarily used in hospital settings, where a fixed payment per patient treatment based on the diagnosis and the expected required resources is reimbursed [42]. In the area of pharmaceuticals SHIs set fixed amounts as maximum reimbursement limit for interchangeable pharmaceuticals to ensure that costs of equivalent drugs are not overpriced. If patients demand a more expensive option that is not covered by the SHI, they can pay the difference out of pocket. It is interesting to note that pharmaceutical companies and medical device manufacturers typically invest many years in research and development to file a patent or obtain market approval for a new drug. Pharmaceutical companies take the full risk concerning research and development and once they succeed, drugs are first introduced as originators with high and protected prices [43] to amortize research and development cost. It takes several years after the market introduction of a new product to reach a break-even point [44]. After a certain time, generics enter the market as copy of the originals and with a lower price. In contrast, DiGA providers have the opportunity to preliminary list their reimbursable mHealth app in the DiGA directory and have their costs already reimbursed by SHI before providing the medical evidence. This represents a significant departure from the existing healthcare system in Germany and it remains open to see whether this part of the DiGA fast-track procedure will still exist in the future. However, the cost structure of DiGAs is different compared to pharmaceuticals since research and development cost are expected to be lower in the beginning but maintenance cost higher during the lifetime [1]. Furthermore, DiGAs do not yet have a standardized process for patenting and thus are expected to be exposed to a greater risk of copying. Hence, we argue that these differences should also be reflected in the pricing model (i.e. lower prices in the beginning, introduction of patenting and price protection periods) and that transparency and mutual understanding of cost structures must be created for both sides to align on fair pricing and payment terms.

### Further development of pricing and payment terms is recommended

We assessed several new pricing strategies for DiGAs since DiGAs represent a new field in standard care with own specifics and thus might require innovative compensation approaches [34]. We assume that these findings can also be transferred to reimbursable mHealth apps in other countries given their expected global growth and the associated need for efficient pricing and payment mechanisms [45–47].

We found that usage dependent pricing models are especially welcomed by SHIs since SHIs only pay for DiGAs that are prescribed and actually used by the patient. According to the GKV-SV only 80% of prescribed DiGAs were actually activated between September 2020–2021 [48]. The concrete operationalization is conceivable in different ways: Payments could be either contingent on the patient receiving an activation code, downloading the app, logging into the app, spending a certain time per day, week or month on the app or receiving a follow-up prescription. Based on this approach, usage could be described as “hygiene factor” that needs to be fulfilled to get a payment. Payers could also define a staggering concept where the percentage paid to the developers depends on the actual usage time (e.g., how many hours per week the app is used). Although possibilities are manyfold, we believe it is key to find a pragmatic measurement that is robust across indications. Other authors suggest introducing daily (instead of quarterly) alternating DiGA prices so that they can also be prescribed for a shorter test period [34].

Representatives of the DiGA providers’ expert group emphasize that it is important to understand that usage is not equal to actual effectiveness of the app and therefore should not be misinterpreted. Since the minimum required usage time varies across indication groups, it might be difficult to determine it in advance. Nevertheless, we believe that app usage can be a proxy for app effectiveness. Moreover, DiGA provider experts highlight that usage based pricing (if implemented for DiGAs) should also be applied to the area of drug management to optimize cost for pharmaceuticals (e.g., drugs that are bought in the pharmacy but never used by the patients).

Furthermore, we introduced a new design choice for pricing models in health care based on our research, namely the user experience based pricing model. This model did not receive much support throughout the expert interviews which might be due to a lack of proof of concept and a lack of an established manipulation-free measurement without neglecting medical effectiveness.

Next, we found that a cost based pricing model might be highly beneficial for DiGA providers since it reduces their financial risk. An often discussed principle in the pharmaceutical industry is the so called two part pricing principle, where payers pay a fixed fee for having access to the drug and a variable price at margin cost for each prescribed unit [49, 50]. The variable part of this model has similarities to the "per prescribed unit" payout condition. A fixed fee independent of the individual SHI size would discriminate smaller SHIs since the fixed costs are distributed among a smaller population of insurees compared to larger SHIs.

Concerning the managed entry agreement based pricing model, we found that SHIs consider the managed entry agreement based pricing model to be significantly more important than DiGA providers. We hypothesize that the difference can be explained by the fact that SHIs have a stronger negotiating power compared to DiGA providers and thus speculate on individual arrangements. Also, this pricing model could help SHIs to differentiate from other SHIs and use it as additional sales argument. The preference of SHIs for the managed entry agreement based pricing model is further highlighted by their perception on the model’s long-term financial sustainability. DiGA providers are, on the contrary, significantly less likely to rate this pricing model as financially sustainable on the long term.

### PROMs as one possibility to operationalize value-based care?

Our expert interviews revealed that the value based care pricing model is suspected to create an incentive structure where patients would benefit the most. A study of Kuck et. al including 89 hospitals across Germany found not only that value based care will promote a more efficient use of health care resources within the health care system but also that value based care could lead to cost savings in general. Nevertheless, the authors stated that the implementation of value based care pricing is one major hurdle [51].

This is in line with our results—although both expert groups supported the idea of value based care pricing—they saw difficulties in implementing this reimbursement system. Different arrangements to address this issue are possible: The patient, the supervising physician or both could evaluate the effectiveness of the DiGA. Nevertheless, experts saw difficulties in having the physician evaluating the application since this might be associated with greater effort and too much work for the prescribing physician. Self-reporting of the app’s effectiveness by patients with indication specific digital questionnaires integrated in the app, is already discussed in literature [19, 52]. The methodology is described as “patient reported outcome measures (PROMs)” which could be one way to operationalize value based care. Payers could define a minimum effectiveness rating (i.e., threshold) which needs to be met by the app in order to be reimbursed by the payer. In addition to patient ratings, Brandt suggests to simultaneously measure the usage of the application to determine whether the DiGA is actually used and therefore track back the positive self-reported effect to the DiGA [19]. In our expert interviews, opinions on PROMs were discussed overall controversial: some experts think PROMs could be an easy operationalization method (“It is like agreeing on service level agreements for a DiGA”—DiGA provider expert 6), while others emphasize the disadvantages of such a model (“Theoretical a good idea, but not measurable in practice”—SHI expert 3). Acknowledged implementation hurdles were: existing data protection regulations, importance of the timing of the measurement, dependence of indication groups, susceptibility to manipulation, the problem about the causality effect of PROMs and the DiGA treatment and the fact that PROMs are not suitable to measure improvements of structural effects [53].

### Refinement of the developed pricing and payment taxonomy for reimbursable mHealth apps through expert input

First, we added the analog equivalent pricing model, which enables a comparison with existing non-digital therapies and thus aims to create a basic understanding for the price level of reimbursable mHealth apps. The expediency of a comparative therapy as price anchor was critically discussed since reimbursable mHealth apps are scalable with lower cost than comparative therapies according to the expert opinions. Furthermore, the definition of a benchmark baseline is challenging because it needs to be defined whether a group versus single therapy is set as the benchmark. Some experts claimed that this price dimension would not incorporate that some reimbursable mHealth apps are designed as additional and not substitutable therapy and therefore should not have the same price level. This thought is in line with existing work on the value of digital health interventions which elaborates that the value of mHealth apps should be specified by the incremental benefit over the current standard of care [54].

The second major change was to differentiate between a real-world-evidence and scientific evidence based value based care. In the case of the real-world-evidence value based care, payment would depend on the actual medical evidence in the field independent of any prior studies or evidence proofs. This result is in line with existing research which states that continuous evaluation is crucial for strategic improvements and sustainable benefit assessment of mHealth apps [5, 55]. Those assessments could either be made by the patients themselves (see PROMs) or by the respective supervising physician. In contrast, scientific evidence based value based care is based on prior clinical evidence through clinical trials.

The third change was adding the representation board of payers as negotiation board. Similar to the current German operating model, the representation board, consisting of multiple payers, would negotiate the price and payment terms which will be applicable for all payers. Compared to the individual health insurance negotiation board, this dimension is on the one side more standardized and hence avoids that mHealth app developers need to sign contracts with individual payers, but on the other side less flexible in terms of individual agreements, e.g. lack of integration with the electronic health record or difficulty of including telemedicine [56].

Existing work focuses on single elements of our taxonomy but to the best of our knowledge there is no structured taxonomy of different pricing options. Hence, our pricing and payment taxonomy for reimbursable mHealth apps provides a comprehensive overview of different dimensions for the design and negotiation of the pricing and payment terms of reimbursable mHealth apps. The taxonomy therefore serves as planning and decision basis for developers, health policy makers and payers. Different design choices can be flexibly combined to design a specific pricing and payment strategy for individual markets making our developed taxonomy internationally applicable.

### Limitations and further research

Due to the study design, our study comes with some limitations. First, the survey period of the expert interviews was with five months rather long and new regulatory guidelines were adjusted in this time period in Germany (see maximum price limit for DiGAs, [10]). One could argue that this could lead to bias in our data but since our interviews aimed to assess opinions and ideas for future pricing models, we are confident that this effect is negligible. Second, we are aware that our sample size per expert group is low for the semi-quantitative analysis and that it is critical to make significant statements. We mitigated this issue by using a significant test that is suitable for small samples [57]. Furthermore, a saturation of content was reached in the qualitative content analysis. Third, the composition of our expert group – especially within the DiGA providers’ expert group, is another limitation of our research. We aimed to include more officially listed DiGA providers but unfortunately were not able to engage a greater number of experts who were willing to participate in our research study. The willingness of DiGA providers to speak on pricing and payment was hence low which might be due to the fact that they do not want to disclose their business models. As we partially used the snowball principle to find participating experts, we are aware that we might have sample selection bias in our underlying data [58]. Nevertheless—as we aimed to create a comprehensive and widely applicable pricing and payment taxonomy for reimbursable mHealth apps, we think it is valuable to include the opinion of further mHealth app developers. Fourth, we only conducted a theoretical evaluation of our pricing and payment taxonomy for reimbursable mHealth apps by an interdisciplinary group of researchers in the last iteration of our taxonomy development process, but we think it would be a valuable research contribution to conduct this evaluation also with practitioners. Hence, further research could build on the pricing and payment taxonomy developed in this paper and evaluate it in practice, e.g. probing the developed taxonomy with representatives of payers, developers or policy makers. Based on the findings on the operationalization of the value based care model, we think that further research should find a pragmatic and easily implementable operationalization method. Overall, we argue that both DiGA providers and SHIs have a strong cost-based mindset and that it requires a mindset shift in Germany with regards to value based care pricing and payment terms. Further research should, in the context of the solidarity principle in Germany, therefore build a health economic model to structurally evaluate the cost use effectiveness of mHealth apps in Germany.

## Conclusions

All in all, we conclude that both expert groups appreciate the introduction of DiGAs in general, but especially SHIs see optimization potential with regards to DiGA pricing. To increase the acceptance of DiGA among SHIs in Germany, a revision of the current pricing and reimbursement model appears necessary. Adjustments to this model could not only enhance DiGA acceptance but potentially lead to higher utilization rates if SHIs actively promote DiGA. We anticipate reimbursable mHealth apps to play a growing role in patient care worldwide increasing the need for a fair pricing model in the future. Countries that are currently considering the implementation of reimbursement models for mHealth apps similar to Germany’s DiGA fast-track procedure can benefit from Germany’s experience presented in this article. They might for example directly implement a value based care model to streamline the adoption process and improve the integration of digital health solutions into their healthcare system. The value based care pricing model was seen equally important by both SHIs and DiGA providers, hence we concluded that a value based care pricing model is expected to bring the greatest benefit for patients in the future. Beyond that, our work not only develops a comprehensive pricing and payment taxonomy for reimbursable mHealth apps, but also identifies differences between pricing and reimbursement models and discusses aspects of applicability. Our developed taxonomy is therefore relevant for both researchers and practitioners. On the one side it can serve as a basis for further scientific mHealth app pricing and reimbursement research. On the other side the taxonomy can serve as decision support for policy makers, payers and mHealth app developers, helping to develop and refine pricing and reimbursement regulations. This will foster wider acceptance for reimbursable mHealth apps enabling them to reach their full potential in the future.

## Supplementary Information

Additional file 1: Interview guideline for expert interviews. The file contains the guiding questions for the expert interviews with representatives of providers of digital health applications and representatives of statutory health insurances.

Additional file 2: Consolidated criteria for reporting qualitative studies (COREQ): 32-item checklist. The file contains the checklist according to [28] Tong et al. to report qualitative research along three domains: Research team and reflexivity, Study design, Analysis and findings.

Additional file 3: Completed PRISMA-ScR (Preferred Reporting Items for Systematic Reviews and Meta-Analyses extension for Scoping Reviews) checklist. The file contains the checklist according to [32] to report scoping reviews along the following dimensions: Title, Abstract, Introduction, Methods, Results, Discussion, Funding.

Additional file 4: Overview of included literature records of first iteration (Scoping review). The file contains a tabular overview of included literature records in the first iteration along the following dimensions: Title, Authors, Journal name, Year of publication, DOI, Research focus, Derived pricing strategy.

## Data Availability

The datasets generated during the current study are available from the corresponding author on reasonable request.

## Abbreviations

App: application
BfArM: German Federal Institute for Drugs and Medical Devices (“Bundesamt für Arzneimittel und Medizinprodukte“)
COREQ: Consolidated criteria for reporting qualitative studies
DiGA: German state-certified digital health application (“Digitale Gesundheitsanwendung”)
DVG: German law introduced in late 2019 aiming for health care digitization (“Digitale-Versorgung-Gesetz”)
EBM: Uniform evaluation standard for medical services in Germany (“Einheitliche Bewertungsmaßstab”)
GKV-SV: Central association of the statutory health insurances ("Spitzenverband der gesetzlichen Krankenkassen")
MCDA: multi criteria decision analysis
mHealth: mobile health
PRISMA-ScR: Preferred reporting items for systematic reviews and meta-analyses – Scoping review extension
PROMs: Patient reported outcome measures
SGB: German law with regulations for statutory health insurances (“Sozialgesetzbuch”)
SHI: Statutory health insurance
