# Supplementary material for: Negotiating pricing and payment terms for insurance covered mHealth apps: a qualitative content analysis and taxonomy development based on a German experience
Source: Health Econ Rev. 2024 Oct 4;14:81. doi: 10.1186/s13561-024-00558-8 (PMC11451222; doi:10.1186/s13561-024-00558-8)
Supplement: Supplementary file 1 — Additional file 1: Interview guideline for expert interviews. The file contains the guiding questions for the expert interviews with representatives of providers of digital health applications and representatives of statutory health insurances. [file 13561_2024_558_MOESM1_ESM.pdf]

# Additional file 1: Interview guideline for expert interviews

*The participants will be informed in detail about the processing of personal data within the scope of the research study by e-mail and sign a consent form.*

The expert interviews are conducted in two groups:

- Expert group 1: Representatives of providers of digital health applications (DiGAs)
- Expert group 2: Representatives of statutory health insurances

The following guiding questions serve as orientation for the expert interview and can be adapted during the interview, if the basic statements are not changed. There are two types of questions: Open ended questions as well as semi-quantitative questions. The expert interview will be structured into four sections:

A) Introduction and discussion of challenges and opportunities of DiGA pricing regulations
B) Discussion of alternative pricing models
C) Evaluation of alternative pricing models
D) Further ideas and forecast of general DiGA development

**Part A: Introduction and discussion of challenges and opportunities of DiGA pricing regulations**

- Can you please introduce yourself briefly? In which area do you work in your company?
- What positive experiences have you had regarding pricing and reimbursement of digital health applications (hereafter referred to as DiGAs), and eventually the fast-track procedure?
- What negative experiences have you had regarding DiGAs' pricing and reimbursement, and eventually the fast-track procedure?
- Who in your organization is responsible for price-related decisions related to DiGAs?
- What are the components of the one-time development cost of DiGAs?
- What are the components of the ongoing operating cost of DiGAs?

**Part B: Discussion of alternative pricing models**

- In a cost-based pricing model, the price of a DiGA is based on the individual development and operating costs of the app. What is your opinion on this pricing model?
- In a reference price-based pricing model, average prices are set per indication group and applied equally to all DiGAs in the respective indication group. What is your opinion on this pricing model?
- In an external reference based pricing model, an international benchmark price/comparison price is derived based on an application already existing in the market. What is your opinion on this pricing model?
- In a value based care pricing model, the price of the app is based on the actual medical benefit for the patient. Payment is only made if the treatment is actually successful. What is your opinion on this pricing model?
- In a usage based pricing model, the price of the app is based on the usage duration per patient. What is your opinion on this pricing model?
- In a user experience based pricing model, the price of the app is based on the average patient ratings in terms of user experience of the app. What is your opinion on this pricing model?
- In a managed entry agreement based pricing model, DiGA providers and SHIs negotiate a framework agreement that is subject to certain conditions. What is your opinion on this pricing model?

**Part C: Evaluation of alternative pricing models**

*Using a pre-structured Excel template, experts' opinions with regard to different evaluation criteria per discussed pricing model will be recorded.*

- Perceived importance of the pricing model: On a scale of 1-5, with 5 being very important, how important do you think the pricing model is?
- Administrative effort to implement the pricing model: How would you rate the administrative effort of implementing this pricing model on a scale of 1-5, with 5 being very high?
- Use for different stakeholders: On a scale of 1-5, with 5 being very high, how would you rate the benefits of this pricing model for the patient, the physician, the DiGA provider and the statutory health insurance? (please estimate separately for each stakeholder)
- Perceived fairness of risk sharing: On a scale of 1-5, with 5 being very high, how would you rate the fairness of risk sharing between DiGA providers and statutory health insurances?
- Balance between undesired and desired effects: On a scale of 1-5, with 5 being very balanced, how would you rate the balance between desired and undesired effects in this pricing model?

- Perceived long-term financial sustainability: How would you rate the long-term financial sustainability of the pricing model on a scale of 1-5, with 5 being very high?
- Ability to predict cost: How would you rate the ability to predict cost with this pricing model on a scale of 1-5, with 5 being very difficult?

**Part D: Further ideas and forecast of general DiGA development**
*This section can be shortened if interview time runs out.*

- Of the pricing models discussed, what are your top three?
- Should pricing models where patients pay some or all of the costs be established?
- What is your perspective on alternative payment models (e.g., startup incubators, innovation funds, strategic partnerships) instead of reimbursement through statutory health insurances?
- Do you have other ideas for novel pricing models?
- How do you think the cost of DiGAs will develop over the next 10 years?
- How do you estimate the short-, medium- and long-term cost effect of DiGAs on the German healthcare system?
- Is there anything else you would like to share on the pricing and reimbursement of DiGAs?
