# Supplementary material for: Negotiating pricing and payment terms for insurance covered mHealth apps: a qualitative content analysis and taxonomy development based on a German experience
Source: Health Econ Rev. 2024 Oct 4;14:81. doi: 10.1186/s13561-024-00558-8 (PMC11451222; doi:10.1186/s13561-024-00558-8)
Supplement: Supplementary file 2 — Additional file 2: Consolidated criteria for reporting qualitative studies (COREQ): 32-item checklist. The file contains the checklist according to [28] Tong et al. to report qualitative research along three domains: Research team and reflexivity, Study design, Analysis and findings. [file 13561_2024_558_MOESM2_ESM.pdf]

Additional file 2: Consolidated criteria for reporting qualitative studies (COREQ): 32-item checklist

Developed from:
Tong A, Sainsbury P, Craig J. Consolidated criteria for reporting qualitative research (COREQ): a 32-item checklist for interviews and focus groups. Int J Qual Health Care 2007; 19(6):349–57

No. Item	Description	Study specifics
**Domain 1: Research team and reflexivity**		
Personal Characteristics		
1. Interviewer/facilitator	Which author/s conducted the interview or focus group?	BF
2. Credentials	What were the researcher's credentials? E.g. PhD, MD	Master of Science
3. Occupation	What was their occupation at the time of the study?	Educational leave from a large international consulting firm
4. Gender	Was the researcher male or female?	Female
5. Experience and training	What experience or training did the researcher have?	Training by SM and LF, prior interview experience
Relationship with participants		
6. Relationship established	Was a relationship established prior to study commencement?	No
7. Participant knowledge of the interviewer	What did the participants know about the researcher? e.g. personal goals, reasons for doing the research	Participants were informed on the purpose of the study via mail, received an information letter regarding the processing of personal data within the scope of the study and gave their consent. Participants were aware that the study was part of a doctoral research project.
8. Interviewer characteristics	What characteristics were reported about the inter viewer/facilitator? e.g. Bias, assumptions, reasons and interests in the research topic	Doctoral student at Chair of Health Care Informatics of Witten/Herdecke University; Research topic is financial perspective of "DiGAs" in Germany; Master of Science in Business Informatics; Prior work for large international consulting firm
**Domain 2: Study design**		
Theoretical framework		
9. Methodological orientation and theory	What methodological orientation was stated to underpin the study? E.g. grounded theory, discourse analysis, ethnography,	Qualitative content analysis

	phenomenology, content analysis	
Participant selection		
10. Sampling	How were participants selected? e.g. purposive, convenience, consecutive, snowball	Purposive & snowball
11. Method of approach	How were participants approached? e.g. face-to-face, telephone, mail, email	Mail, LinkedIn and/or telephone
12. Sample size	How many participants were in the study?	69 potential experts contacted in total (incl. snowball)
13. Non-participation	How many people refused to participate or dropped out? Reasons?	53, hypothesis was that potential experts lack the time for an interview or do not want to disclose business model/ strategic pricing decisions etc.
Setting		
14. Setting of data collection	Where was the data collected? e.g. home, clinic, workplace	Remotely
15. Presence of non-participants	Was anyone else present besides the participants and researchers?	No
16. Description of sample	What are the important characteristics of the sample? e.g. demographic data, date	16 experts, data collected between May and September 2022
Data collection		
17. Interview guide	Were questions, prompts, guides provided by the authors? Was it pilot tested?	See supplementary material 1, first interview served as pilot, data was included in the results
18. Repeat interviews	Were repeat interviews carried out? If yes, how many?	No
19. Audio/visual recording	Did the research use audio or visual recording to collect the data?	Yes, all interviews were recorded
20. Field notes	Were field notes made during and/or after the interview or focus group?	Yes, memos were written during interview transcriptions
21. Duration	What was the duration of the inter views or focus group?	30-90 minutes per interview
22. Data saturation	Was data saturation discussed?	Not as part of the interviews
23. Transcripts returned	Were transcripts returned to participants for comment and/or correction?	No

**Domain 3: Analysis and findings**		
Data analysis		
24. Number of data coders	How many data coders coded the data?	1
25. Description of the coding tree	Did authors provide a description of the coding tree?	No
26. Derivation of themes	Were themes identified in advance or derived from the data?	Themes were derived in advance (first coding cycle) and from the data (second coding cycle)
27. Software	What software, if applicable, was used to manage the data?	Trint & MAXQDA
28. Participant checking	Did participants provide feedback on the findings?	No
Reporting		
29. Quotations presented	Were participant quotations presented to illustrate the themes/findings? Was each quotation identified? e.g. participant number	Yes, quotes identified per expert group
30. Data and findings consistent	Was there consistency between the data presented and the findings?	Yes
31. Clarity of major themes	Were major themes clearly presented in the findings?	Yes
32. Clarity of minor themes	Is there a description of diverse cases or discussion of minor themes?	Yes partially
