# Supplementary material for: Negotiating pricing and payment terms for insurance covered mHealth apps: a qualitative content analysis and taxonomy development based on a German experience
Source: Health Econ Rev. 2024 Oct 4;14:81. doi: 10.1186/s13561-024-00558-8 (PMC11451222; doi:10.1186/s13561-024-00558-8)
Supplement: Supplementary file 3 — Additional file 3: Completed PRISMA-ScR (Preferred Reporting Items for Systematic Reviews and Meta-Analyses extension for Scoping Reviews) checklist. The file contains the checklist according to [32] to report scoping reviews along the following dimensions: Title, Abstract, Introduction, Methods, Results, Discussion, Funding. [file 13561_2024_558_MOESM3_ESM.pdf]

Additional file 3: Completed PRISMA-ScR (Preferred Reporting Items for Systematic Reviews and Meta-Analyses extension for Scoping Reviews) checklist

Developed from:
Tricco AC, Lillie E, Zarin W, O'Brien KK, Colquhoun H, Levac D et al. PRISMA Extension for Scoping Reviews (PRISMA-ScR): Checklist and Explanation. Ann Intern Med 2018; 169(7):467–73.

SECTION	ITEM	PRISMA-ScR CHECKLIST ITEM	REPORTED IN SECTION
**TITLE**			
Title	1	Identify the report as a scoping review.	Not applicable since scoping review was one part of the overarching methodology.
**ABSTRACT**			
Structured summary	2	Provide a structured summary that includes (as applicable): background, objectives, eligibility criteria, sources of evidence, charting methods, results, and conclusions that relate to the review questions and objectives.	**Background:** The pricing of digital health applications in Germany ("DiGA") which are fully reimbursable by statutory health insurances has faced criticism from insurers. In the first year, DiGA providers are allowed to set the price for their application freely, even if clinical evidence of effectiveness is not yet available, as long as certain regulatory conditions are met.<br><br>**Objectives:** Due to the demand for a revision of the DiGA pricing model, this scoping review is intended to provide an overview of possible alternative pricing strategies that exist in other areas of healthcare. The already existing pricing strategies are intended to be used as a base for the development of a new comprehensive taxonomy for the design and negotiation of pricing and payment terms of reimbursable mHealth apps.<br><br>**Eligibility criteria and methods:** A scoping review was conducted to identify relevant research studies presenting pricing strategies for mobile health applications and

			pharmaceuticals. The ‘PubMed’ database was searched with a keyword search. Eligibility criteria were that the article was published between 1.3.2017 and 28.02.2022, was written in German or English language, an abstract was available and that the article had a focus on pricing strategies. The scoping review was supplemented by both backward and forward snowballing.<br><br>**Results:** Of 806 records initially identified through the keyword and snowball search, 32 articles were eligible. We found 6 pricing strategies throughout the scoping review: cost based pricing model, reference price based pricing model, external reference based pricing model, value based care pricing model, usage based pricing model, managed entry agreement based pricing model. While most of the articles mentioned one single pricing model, three articles discussed two and three pricing strategies and one article four pricing strategies. Value based care pricing models were mentioned most frequently (22/32, 69%) followed by external reference based pricing (7/32, 22%) and reference price based pricing models (5/32, 16%).<br><br>**Conclusions:** Overall, the scoping review was a suitable approach to identify new pricing models in the context of DiGA. Value based care pricing models are frequently discussed in literature, but not yet arrived in medical practice. It would be interesting to analyze the opinions of experts from statutory health insurances and DiGA providers whether the model is suitable for DiGA in practice.
**INTRODUCTION**			
Rationale	3	Describe the rationale for the review in the context of what is already known. Explain why the review questions/objectives lend themselves to a scoping review approach.	Germany was the first country worldwide to offer digital health applications that are fully reimbursable by statutory health insurances. The current pricing model, which allows DiGA providers to freely set the price during the first year

			within certain regulatory conditions, is unique. Representative associations of Germany's statutory health insurances have expressed in various white papers that adjustments to this pricing model are necessary. A scoping review was identified as suitable method to gain a broader understanding of alternative pricing models for mobile health apps or pharmaceuticals within the scientific community. The results of this analysis serve as a starting point for discussions with experts from DiGA providers and statutory health insurance organizations as part of a comprehensive taxonomy development process.
Objectives	4	Provide an explicit statement of the questions and objectives being addressed with reference to their key elements (e.g., population or participants, concepts, and context) or other relevant key elements used to conceptualize the review questions and/or objectives.	Our review aimed at identifying potential different pricing and reimbursement strategies in the area of mobile health apps or pharmaceuticals.
**METHODS**			
Protocol and registration	5	Indicate whether a review protocol exists; state if and where it can be accessed (e.g., a Web address); and if available, provide registration information, including the registration number.	As this review was part of a more comprehensive study approach, we did not prepare, register, and publish a review protocol.
Eligibility criteria	6	Specify characteristics of the sources of evidence used as eligibility criteria (e.g., years considered, language, and publication status), and provide a rationale.	To be included in the scoping review, articles must be listed in the 'PubMed' database and published between 01.03.2017 and 28.02.2022 to account for more recent research findings. The article must be in English or German language and must include an abstract to ensure a high level of quality and guarantee that no language barriers hinder the comprehensibility. The article types 'clinical trials' and 'randomized controlled trials' were excluded from the analysis since their main focus lies on the proof of effectiveness and security of new treatment options rather than suggestions for new pricing and reimbursement schemes. Abstract and full-text assessment was then

			conducted to filter those articles that incorporated pricing strategies (see also Figure 3).
Information sources	7	Describe all information sources in the search (e.g., databases with dates of coverage and contact with authors to identify additional sources), as well as the date the most recent search was executed.	The ’PubMed’ database was the underlying source for the scoping review. The search query was performed on 28.02.2022.
Search	8	Present the full electronic search strategy for at least 1 database, including any limits used, such that it could be repeated.	The following search string was used in the ‘PubMed’ database keyword search:<br>(pricing strategies[Title/Abstract] OR pricing strategy[Title/Abstract] OR pricing[Title/Abstract] OR reimbursement[Title/Abstract] OR payment[Title/Abstract]) AND (mHealth apps[Title/Abstract] OR DiGA[Title/Abstract] OR digital health applications[Title/Abstract] OR pharmaceuticals[Title/Abstract])<br>We discussed and aligned the search string among all authors.
Selection of sources of evidence	9	State the process for selecting sources of evidence (i.e., screening and eligibility) included in the scoping review.	Please refer to Figure 3
Data charting process	10	Describe the methods of charting data from the included sources of evidence (e.g., calibrated forms or forms that have been tested by the team before their use, and whether data charting was done independently or in duplicate) and any processes for obtaining and confirming data from investigators.	All included articles were recorded in a tabular overview including title, authors, journal name, year of publication, DOI, research focus and derived pricing strategy (please refer to additional file 4 for the overview).
Data items	11	List and define all variables for which data were sought and any assumptions and simplifications made.	The following data items were coded as part of the scoping review: title of the article, authors, journal name, year of publication, DOI, research focus, derived pricing strategy. (please refer to additional file 4 for the overview)
Critical appraisal of individual sources of evidence	12	If done, provide a rationale for conducting a critical appraisal of included sources of evidence; describe the methods used and how	We did not conduct a critical appraisal of individual sources of evidence since we aimed to evaluate the results of the scoping review with both researchers and experts from

		this information was used in any data synthesis (if appropriate).	statutory health insurances and DiGA providers as part of our comprehensive taxonomy development process. Through each iteration throughout the taxonomy development, we have supplemented, critically examined and sharpened our results.
Synthesis of results	13	Describe the methods of handling and summarizing the data that were charted.	All articles included in the scoping review were recorded in a tabular overview including title of the article, authors, journal name, year of publication, DOI, research focus, and derived pricing strategy.
**RESULTS**			
Selection of sources of evidence	14	Give numbers of sources of evidence screened, assessed for eligibility, and included in the review, with reasons for exclusions at each stage, ideally using a flow diagram.	Please refer to Figure 3
Characteristics of sources of evidence	15	For each source of evidence, present characteristics for which data were charted and provide the citations.	Please refer to Figure 3 and Additional file 4
Critical appraisal within sources of evidence	16	If done, present data on critical appraisal of included sources of evidence (see item 12).	Not applicable
Results of individual sources of evidence	17	For each included source of evidence, present the relevant data that were charted that relate to the review questions and objectives.	Please refer to Additional file 4
Synthesis of results	18	Summarize and/or present the charting results as they relate to the review questions and objectives.	Please refer to Additional file 4
**DISCUSSION**			
Summary of evidence	19	Summarize the main results (including an overview of concepts, themes, and types of evidence available), link to the review questions and objectives, and consider the relevance to key groups.	Our scoping review revealed 6 pricing strategies: cost based pricing model, reference price based pricing model, external reference based pricing model, value based care pricing model, usage based pricing model, managed entry agreement based pricing model.
Limitations	20	Discuss the limitations of the scoping review process.	Our defined eligibility criteria might be a limitation of the results of the scoping review. First, we limited our search to recent articles published between 1.3.2017 and 28.2.2022

			(5 year time horizon), i.e. we might have missed older articles. Second, we limited our search to English and German articles and excluded clinical trials and randomized controlled trials. Third, we limited our scoping review to only one database and could have searched several databases to include as many articles as possible. However, the 'PubMed' database was carefully chosen and offers a reliable source for articles in the healthcare and medical sector, and we supplemented it with a snowball search. Since our scoping review was only one part of a comprehensive taxonomy development process and served as first basis to get an overview on existing pricing strategies in the latest scientific discussions, the scoping review was solely conducted by one author, but validated in the next iteration by researchers and practitioners.
Conclusions	21	Provide a general interpretation of the results with respect to the review questions and objectives, as well as potential implications and/or next steps.	The 32 articles included in the review highlight various pricing strategies discussed in academic literature. The review demonstrates that a wide range of alternative pricing strategies is available and could potentially be applied for DiGA. The identified pricing strategies provide the foundation for the next cycle of our taxonomy development, where we validated the findings through an interdisciplinary scientist panel.
**FUNDING**			
Funding	22	Describe sources of funding for the included sources of evidence, as well as sources of funding for the scoping review. Describe the role of the funders of the scoping review.	No funding was received for this research. Bettina Freitag received a doctoral scholarship granted by Ev. Studienwerk Villigst.
