# Supplementary material for: Negotiating pricing and payment terms for insurance covered mHealth apps: a qualitative content analysis and taxonomy development based on a German experience
Source: Health Econ Rev. 2024 Oct 4;14:81. doi: 10.1186/s13561-024-00558-8 (PMC11451222; doi:10.1186/s13561-024-00558-8)
Supplement: Supplementary file 4 — Additional file 4: Overview of included literature records of first iteration (Scoping review). The file contains a tabular overview of included literature records in the first iteration along the following dimensions: Title, Authors, Journal name, Year of publication, DOI, Research focus, Derived pricing strategy. [file 13561_2024_558_MOESM4_ESM.pdf]

Additional file 4: Overview of included literature records of first iteration (Scoping review)

Title	Authors	Journal name	Year of publication	DOI	Research focus	Derived pricing strategy
The new approval process for the reimbursement of digital health applications (DiGA) from the perspective of the German statutory health insurance	Gregor-Haack J, Busse T, Hagenmeyer EG	Bundesgesund-heitsblatt	2021	https://doi.org/10.1007/s00103-021-03401-1	Evaluation of initial six months of the DiGA fast-track procedure in Germany; highlighting the concerns of statutory health insurances regarding insufficient proof of medical effectiveness, economic efficiency and the need for price regulation adjustments	Reference price based
Assessing the pricing and benefits of oncology products: an update	Petrou P	Expert review of pharmaco-economics & outcomes research	2021	10.1080/14737167.2021.1926987	Examination of the rising oncology pharmaceutical expenditure highlighting the need for new reimbursement schemes	Value based care, Managed entry agreement based
Comparative Approaches to Drug Pricing	Kang SY, Bai G, DiStefano MJ, Socal MP, Yehia F, Anderson GF	Annual review of public health	2019	https://doi.org/10.1146/annurev-publhealth-040119-094305	Assessment of drug pricing mechanisms in the USA and comparison to the mechanisms in the United Kingdom, Australia, Germany, France & Japan	External reference based, Value based care
Pricing of in-patent pharmaceuticals in the Middle East and North Africa: Is external reference pricing implemented optimally?	Kanavos P, Kamphuis BW, Fontrier AM, Colville Parkin G, Saleh S, Akhras KS	Health Policy	2020	https://doi.org/10.1016/j.healthpol.2020.07.017	Comparison of pharmaceutical pricing policies in the Middle East and North African region with focus on external reference pricing	External reference based
External Reference Pricing for Pharmaceuticals-A Survey and Literature Review to Describe Best Practices for Countries With Expanding Healthcare Coverage	Holtorf AP, Gialama F, Wijaya KE, Kaló Z	Value in Health Regional Issues	2019	https://doi.org/10.1016/j.vhri.2019.04.003	Presentation of best practices for implementing external reference pricing for pharmaceuticals	External reference based

Health technology assessment in Japan: a work in progress	Kamae I, Thwaites R, Hamada A, Fernandez JL	Journal of Medical Economics	2019	10.1080/13696998.2020.1716775	Assessment of the Health Technology Assessment program in Japan for selected pharmaceuticals and medical devices	Value based care
Risk-sharing schemes to finance expensive pharmaceuticals: Interdisciplinary analyses	Buch C, Schildmann J, Zerth J	Wellcome Trust	2021	NBK585090	Analysis of performance- or outcome-based risk-sharing agreements including case study of multiple sclerosis drug in the United Kingdom	Value based care
Does external reference pricing deliver what it promises? Evidence on its impact at national level	Kanavos P, Fontrier AM, Gill J, Efthymiadou O	The European Journal of Health Economics	2019	https://doi.org/10.1007/s10198-019-01116-4	Assessment of the evidence on external reference pricing and impact on health policy objectives	External reference based
Towards a new Vision for Shared Responsibility in Pharmaceutical Pricing, Coverage and Reimbursement: Policy approaches building on principles of solidarity, transparency and sustainability	Docteur E	Oslo Medicines Initiative	2022	NBK587840	Evaluation of different policy solutions to improve access to high-priced medicines	Managed entry agreement based
Performance-based risk-sharing arrangements for devices and diagnostics in the United States: a systematic review	Chen Y, Carlson JJ	Journal of Managed Care & Specialty Pharmacy	2022	10.18553/jmcp.2022.28.1.78	Review of performance-based risk-sharing arrangements for diagnostics and devices in the United States	Value based care
An assessment of innovative pricing schemes for the communication of value: is price discrimination and two-part pricing a way forward?	Hertzman P, Miller P, Tolley K	Expert review of pharma-coeconomics & outcomes research	2018	https://doi.org/10.1080/14737167.2018.1411192	Discussion of innovative financial- and performance-based pricing schemes for expensive medicines	Cost based, Reference price based, Value based care, Usage based

Variations in external reference pricing implementation: does it matter for public policy?	Gill J, Fontrier AM, Kyriopoulos D, Kanavos P	The European Journal of Health Economics	2019	https://doi.org/10.1007/s10198-019-01100-y	Comparison of different external reference pricing implementations of pharmaceuticals in several countries	External reference based
Effectiveness of National Pricing Policies for Patent-Protected Pharmaceuticals in the OECD: A Systematic Literature Review	Wettstein DJ, Boes S	Applied Health Economics and Health Policy	2019	10.1007/s40258-018-0437-z	Review on the effectiveness of national pricing regulations for patent-protected prescription pharmaceuticals in OECD countries	Reference price based, External reference based, Value based care
Drug Treatment Value in a Changing Oncology Landscape: A Literature and Provider Perspective	Frois C, Howe A, Jarvis J, Grice K, Wong K, Zacker C, Sasane R	Journal of Managed Care & Specialty Pharmacy	2019	10.18553/jmcp.2019.25.2.246	Analysis of healthcare system in the United States in terms of a value-based reimbursement model, particularly in oncology from a provider's perspective	Value based care
Barriers for Access to New Medicines: Searching for the Balance Between Rising Costs and Limited Budgets	Godman B, Bucsics A, Vella Bonanno P, Oortwijn W, Rothe CC et al.	Frontiers in Public Health	2018	10.3389/fpubh.2018.00328	Examination of innovative models to manage rising costs of new medicines given budgetary pressure	Reference price based, Value based care, Managed entry agreement based
International impact of external reference pricing: should national policy-makers care?	Fontrier AM, Gill J, Kanavos P	The European Journal of Health Economics	2018	https://doi.org/10.1007/s10198-019-01083-w	Review of external reference pricing models and analysis of pre-defined endpoints	External reference based
Are Value-Based Arrangements the Answer We've Been Waiting for?	Dubois RW, Westrich K, Buelt L	Value in Health	2020	https://doi.org/10.1016/j.jval.2019.10.016	Analysis of value-based care arrangements for therapies in the United States	Value based care
The economics of alternative payment models for pharmaceuticals	Hlávka JP, Yu JC, Goldman DP, Lakdawalla DN	The European Journal of Health Economics	2021	10.1007/s10198-021-01274-4	Comparison of uniform pricing for pharmaceuticals with value-based pricing	Value based care
Value in Health: How It Is Defined and Used in Priority Setting and Pricing in Norway	Melberg HO	Healthcare Papers	2019	10.12927/hcpap.2019.25929	Analysis of a value-based care concept for pharmaceuticals in Norway	Value based care

Innovative Contracting for Pharmaceuticals and Medicaid's Best-Price Rule	Sachs R, Bagley N, Lakdawalla DN	Journal of Health Politics, Policy and Law	2018	https://doi.org/10.1215/03616878-4249796	Discussion of alternative pricing strategies in the area of pharmaceuticals	Reference price based, Value based care
Paying For Value From Costly Medical Technologies: A Framework For Applying Value-Based Payment Reforms	Lopez MH, Daniel GW, Fiore NC, Higgins A, McClellan MB	Health Affairs	2020	https://doi.org/10.1377/hlthaff.2019.0077	Framework development for value-based payment models for medical products including pharmaceuticals, devices and diagnostic tools	Value based care
AMCP Partnership Forum: Improving Quality, Value, and Outcomes with Patient-Reported Outcomes	AMCP Partnership Forum	Journal of Managed Care & Specialty Pharmacy	2018	https://doi.org/10.18553/jmcp.2018.17491	Analysis of challenges and solutions of the implementation of patient-reported outcome measures in healthcare with emphasis on their role in enhancing patient-centered care	Value based care
Using the Delphi method to identify meaningful and feasible outcomes for pharmaceutical value-based contracting	Swart ECS, Parekh N, Daw J, Manolis C, Good CB, Neilson LM	Journal of Managed Care & Specialty Pharmacy	2020	https://doi.org/10.18553/jmcp.2020.26.11.1385	Assessment of using the Delphi surveying technique to align the interest of key stakeholders in the design of value-based pharmaceutical contracts	Value based care
Affordability Challenges to Value-Based Pricing: Mass Diseases, Orphan Diseases, and Cures	Danzon PM	Value in Health	2018	https://doi.org/10.1016/j.jval.2017.12.018	Examination of value-based pricing in the context of chronic, progressive disease, cures and orphan drugs	Value based care
Abandoning List Prices In Medicaid Drug Reimbursement Did Not Affect Spending	Ippolito B, Levy JF, Anderson GF	Health Affairs	2020	10.1377/hlthaff.2019.01354	Evaluation of list price-based reimbursement to invoice-based models in the area of pharmaceuticals	Cost based
Beyond The High Prices Of Prescription Drugs: A Framework To Assess Costs, Resource Allocation, And Public Funding	Darrow JJ, Light DW	Health Affairs	2021	10.1377/hlthaff.2020.00328	Framework development for legislators and scientists to assess total societal cost of drugs	Value based care

Patient Support Program Increased Medication Adherence with Lower Total Health Care Costs Despite Increased Drug Spending	Brixner D, Rubin DT, Mease P, Mittal M, Liu H, Davis M, Ganguli A, Fendrick AM	Journal of Managed Care & Specialty Pharmacy	2019	10.18553/jmcp.2019.18443	Real world evidence on patient support programs for a biology therapy	Value based care
A new pricing strategy evaluation model	Baker T, Collier D, Jayaraman V	International Journal of Operational Research	2017	https://doi.org/10.1504/IJOR.2017.084357	Development of a new pricing measure with the goal of increasing the return on investment	Cost based
Healthcare Digitalization and Pay-For-Performance Incentives in Smart Hospital Project Financing	Moro Visconti R, Morea D	International Journal of Environmental Research and Public Health	2020	10.3390/ijerph17072318	Assessment of impact of healthcare digitization on healthcare infrastructural investments	Value based care
Value-Based Pricing and Reimbursement in Personalised Healthcare: Introduction to the Basic Health Economics	Garrison LP, Towse A	Journal of Personalized Medicine	2017	10.3390/jpm7030010	Evaluation of relationship between value-based pricing and reimbursement in personalized healthcare	Value based care
Beyond validation: getting health apps into clinical practice	Gordon WJ, Landman A, Zhang H, Bates DW	Npj Digital Medicine	2020	https://doi.org/10.1038/s41746-019-0212-z	Framework development of integrating health apps into clinical practice routine	Usage based
A review of international coverage and pricing strategies for personalized medicine and orphan drugs	Degtiar I	Health Policy	2017	https://doi.org/10.1016/j.healthpol.2017.09.005	Review of pricing strategies for personalized medicine and orphan drugs	Cost based, Value based care, Managed entry agreement based
